# Network analysis of the cerebrospinal fluid proteome reveals shared and unique differences between sporadic and familial forms of amyotrophic lateral sclerosis

**DOI:** 10.1186/s13024-025-00838-9

**Published:** 2025-05-15

**Authors:** Adam N. Trautwig, Edward J. Fox, Eric B. Dammer, Anantharaman Shantaraman, Lingyan Ping, Duc M. Duong, Caroline M. Watson, Fang Wu, Seneshaw Asress, Qi Guo, Allan I. Levey, James J. Lah, Federico Verde, Alberto Doretti, Antonia Ratti, Nicola Ticozzi, Cindy V. Ly, Timothy M. Miller, Mark A. Garret, James D. Berry, Eleanor V. Thomas, Christina N. Fournier, Zachary T. McEachin, Nicholas T. Seyfried, Jonathan D. Glass

**Affiliations:** 1 Department of Neurology, Emory University School of Medicine, Atlanta, GA USA; 2 Center for Neurodegenerative Disease, Emory University School of Medicine, Atlanta, GA USA; 3 Department of Biochemistry, Emory University School of Medicine, Atlanta, GA USA; 4 Department of Neurology, Washington University, St Louis, MO USA; 5 Department of Neuroscience, IRCCS Instituto Auxologico Italiano, Milan, Italy; 6 Department of Pathophysiology and Transplantation, Universitá Degli Studi Di Milano, Milan, Italy; 7 Department of Medical Biotechnology and Translational Medicine, Universitá Degli Studi Di Milano, Milan, Italy; 8 Sean M. Healey & AMG Center for ALS, Massachusetts General Hospital, Boston, MA USA; 9 Department of Human Genetics, Emory University School of Medicine, Atlanta, GA USA

**Keywords:** Amyotrophic Lateral Sclerosis ALS, C9orf72, SOD1, Cerebrospinal Fluid (CSF), Weighted Gene Co-Expression Network Analysis (WGCNA), Differentially Abundant Proteins (DAP)

## Abstract

**Background:**

Amyotrophic Lateral Sclerosis (ALS), a neurodegenerative disease involving loss of motor neurons, typically results in death within 3–5 years of disease onset. Although roughly 10% of cases can be linked to a specific inherited mutation (e.g., C9orf72 hexanucleotide repeat expansion or SOD1 mutation), the cause(s) of most cases are unknown. Consequently, there is a critical need for biomarkers that reflect disease onset and progression across ALS subgroups.

**Methods:**

We employed tandem mass tag mass spectrometry (TMT-MS) based proteomics on cerebrospinal fluid (CSF) to identify and quantify 2105 proteins from sporadic, C9orf72, and SOD1 ALS patients, asymptomatic C9orf72 expansion carriers, and controls (*N* = 101). To verify trends in our Emory University cohort we used data-independent acquisition (DIA-MS) on an expanded, four center cohort. This expanded cohort of 259 individuals included 50 sporadic ALS (sALS), 43 C9orf72 ALS, 22 SOD1 ALS, 72 asymptomatic gene carriers (59 C9orf72 and 13 SOD1) and 72 age-matched controls. We identified 2330 proteins and used differential protein abundance and network analyses to determine how protein profiles vary across disease subtypes in ALS CSF.

**Results:**

Differential abundance and co-expression network analysis identified proteomic differences between ALS and control, as well as differentially abundant proteins between sporadic, C9orf72 and SOD1 ALS. A panel of proteins differentiated forms of ALS that are indistinguishable in a clinical setting. An additional panel differentiated asymptomatic from symptomatic C9orf72 and SOD1 mutation carriers, marking a pre-symptomatic proteomic signature of genetic forms of ALS. Leveraging this large, multicenter cohort, we validated our ALS CSF network and identified ALS-specific proteins and network modules.

**Conclusions:**

This study represents a comprehensive analysis of the CSF proteome across sporadic and genetic causes of ALS that resolves differences among these ALS subgroups and also identifies proteins that distinguish symptomatic from asymptomatic gene carriers. These new data point to varying pathogenic pathways that result in an otherwise clinically indistinguishable disease.

**Supplementary Information:**

The online version contains supplementary material available at 10.1186/s13024-025-00838-9.

## Introduction

Amyotrophic Lateral Sclerosis (ALS) is a heterogeneous motor neuron disease that typically results in death within 3–5 years [1, 2]. Clinical manifestations include a spectrum of upper and lower motor neuron involvement and wide variability in disease progression. Roughly 10% of ALS cases are driven by an inherited mutation, of which the most common are C9orf72 hexanucleotide repeat expansion [3, 4] and point mutations in the gene for superoxide dismutase 1 (SOD1) [5]. Though the pathogenic mechanisms underlying the various genetic forms of ALS are likely to be distinct from sporadic disease, the clinical presentations are remarkably similar, making the discovery of biomarkers that distinguish the various forms of ALS of paramount importance. Also, in people who harbor disease-causing mutations but remain asymptomatic, comparison of biomarkers from the pre- and post-symptomatic phase will provide insight into potential markers of disease transition, and also mark an early time point when disease modifying therapies could be started. A focus on cerebrospinal fluid (CSF) biomarkers allows for interrogation of CNS protein changes that may differentiate disease pathways among sporadic and genetic forms of ALS, TDP- 43 and SOD1 pathologies, as well as provide tools allowing for early diagnosis and monitoring of disease activity. Here, our objective was to evaluate the CSF proteome to enhance our comprehension of both shared and distinct disease alterations associated with CNS cell-types and pathways across sporadic and genetic ALS subgroups.

Mass spectrometry-based proteomics coupled with systems biology approaches using co-expression network analysis is a valuable tool for discovery of disease biomarkers and pathways, including in ALS and Alzheimer’s Disease [6–10]. Unbiased proteomics of human brain and CSF coupled with network analysis has emerged as a method for organizing proteome-wide expression data into groups or “modules” of highly correlated proteins that reflect various biological functions linked to neurodegeneration [11]. While ALS brain proteomic networks have been examined [6], ALS CSF proteomic networks from large cohorts that include sporadic and familial ALS across different mutations, and asymptomatic carriers, are under-investigated.

To achieve this, we conducted unbiased proteomics on cerebrospinal fluid (CSF) samples from sporadic ALS (sALS), C9orf72 ALS, C9orf72 asymptomatic carriers, SOD1 ALS, SOD1 asymptomatic carriers, and healthy controls. This analysis was carried out across two datasets using two complementary proteomic approaches, tandem mass tag spectrometry (TMT-MS) and data-independent acquisition mass spectrometry (DIA-MS). Network modules that showed significant changes with ALS subtypes were linked to specific biological processes, including modules associated with the extracellular matrix and heparin binding, cytoskeleton and microglia, and ubiquitination and gluconeogenesis. Many of these protein changes differentiated symptomatic and asymptomatic carriers of disease-causing ALS mutations. Collectively our findings suggest that while sporadic and genetic forms of ALS display largely overlapping CSF proteomes, differences point to unique pathogenic pathways.

## Materials and methods

### Patient cohorts

An initial cohort of ALS spinal fluids was collected from a single center at Emory University in Atlanta, Georgia, USA. This cohort was made up of samples from C9orf72 ALS (*n* = 10), C9orf72 asymptomatic carriers (*n* = 6), SOD1 ALS (*n* = 6), and sALS (*n* = 35), as well as age matched healthy controls (*n* = 44). Characteristics of the Emory cohort are summarized in Table 1 and detailed in Supplemental Table 1. An expanded multicenter cohort was organized adding samples provided by investigators at Massachusetts General Hospital (*n* = 84), Washington University in St. Louis (*n* = 26), and the University of Milan (*n* = 31), and included the initial Emory CSF cohort with an additional 17 samples. This expanded cohort included CSF from 259 individuals: C9orf72 ALS (*n* = 43), C9orf72 asymptomatic carriers (*n* = 59), SOD1 ALS (*n* = 22), SOD1 asymptomatic carriers (*n* = 13), sALS (*n* = 50), and age-matched controls (*n* = 72). Characteristics of the expanded cohort are summarized in Table 2 and detailed in Supplemental Table 2. All CSF samples were collected as part of ongoing research protocols approved by the respective institutions with appropriate patient informed consent.

**Table 1:** Characteristics of the Emory ALS cohort (i.e. Set 1)

	**Control**	**C9orf72 Asymptomatic**	**C9orf72 ALS**	**SOD1 ALS**	**Sporadic ALS**
**Sample number (n)**	44	6	10	6	35
**Site onset**	-	-	5 B, 3 LE, 2 UE	3 LE, 2 U, 1 UE	3 B, 17 LE, 15 UE
**Sex**	27 F, 17 M	2 F, 4 M	4 F, 6 M	2 F, 4 M	13 F, 22 M
**Race**	44 C	5 C, 1H/L	10 C	6 C	3 AA, 1 A, 31 C
**Age at Onset (y)** **(± SD)**	-	-	56.4 (± 8.9)	58.3 (± 4.7)	53.9 (± 11.5)
**Age at Sample (y)** **(± SD)**	64.1 (± 7.6)	51.5 (± 18.5)	57.2 (± 8.7)	59.3 (± 4.6)	56.7 (± 10.9)
**Duration of disease (mo)**^a^** (± SD)**	-	-	41 (± 26)	-	38 (± 26)

Unabridged cohort traits are enumerated in Supplemental Table 1

*Abbreviations F* Female, *M* Male, *AA* African American, *A* Asian, *C* Caucasian, *H/L* Hispanic/Latino, *B* Bulbar, *LE* Lower Extremity, *U* Unknown, *UE* Upper Extremity, *y* Years, *mo* Months

“^a^”10/10 C9orf72 ALS deceased, 19/34 sALS deceased. 2/6 SOD1 ALS deceased, average not calculated

**Table 2:** Characteristics of the Expanded ALS cohort (i.e. Multicenter cohort)

	**Control**	**C9orf72 Asymptomatic**	**C9orf72 ALS**	**SOD1 Asymptomatic**	**SOD1 ALS**	**Sporadic ALS**
Sample number (n)	72	59	43	13	22	50
Center	54 E, 18 MGH	6 E, 53 MGH	10 E, 18 I, 15 W	13 MGH	6 E, 13 I, 3 W	42 E, 8 W
Sex	41 F, 31 M	34 F, 25 M	20 F, 23 M	9 F, 4 M	7 F, 15 M	22 F, 28 M
Race	66 C, 4 AA, 2 A	58 C, 1H/L	10 C, 33 U	13 C	6 C, 16 U	1 A, 3 AA, 37 C, 9 U
Age at sample (y) (± SD)	59.4 (± 12.5)	44.8 (± 12.8)	58.0 (± 7.0)	54.3 (± 12.0)	54.6 (± 12.2)	58.4 (± 10.8)

Unabridged cohort traits are enumerated in Supplemental Table 2

*Abbreviations*: *E* Emory University, *I* Universitá degli Studi di Milano, *MGH* Massachusetts General Hospital, *W* Washington University *F* Female, *M* Male, *AA* African American, *A* Asian, *C* Caucasian, *H/L* Hispanic/Latino, *y* Years, *U* Unknown

### Protein digestion and Tandem Mass Tag (TMT) labeling of CSF

In order to sample the CSF in an unbiased manner and given that we have previously shown that immunodepletion resulted in only a marginal improvement in proteomic coverage, the CSF samples were not immunodepleted prior to digestion [12–14]. First, 50 μL of CSF was transferred to 1 mL deep well plates for digestion with lysyl endopeptidase (LysC) and trypsin. The samples were then reduced and alkylated with 1 μL of 0.5 M tris- 2(-carboxyethyl)-phosphine (ThermoFisher) and 5 μL of 0.4 M chloroacetamide in a 90 °C water bath for 10 min followed with 5 min bath sonication. After the sample was cooled on ice, 56 μL of 8 M urea buffer (8 M urea, 10 mM Tris, 100 mM NaH_2_PO_4_, pH 8.5) with 12.5 mAU of LysC (Wako), was added to each sample, resulting in a final urea concentration of 4 M.

Samples were then mixed well, gently spun down, and incubated overnight at 25 °C for digestion with LysC. The following day, samples were diluted to 1 M urea with a mixture of 360 μL of 50 mM ammonium bicarbonate [15] and 5 μg of Trypsin (ThermoFisher). The samples were subsequently incubated overnight at 25 °C for digestion with trypsin. The next day, the digested peptides were acidified to a final concentration of 1% formic acid and 0.1% trifluoroacetic acid. This was immediately followed by desalting on 30 mg HLB columns (Waters) and then eluted with 1 mL of 50% acetonitrile (ACN) as previously described [16]. To normalize protein quantification across batches, 150 μl of elution was taken from all CSF samples and then combined to generate a pooled sample as previously described [16]. This pooled sample was split into 850 μL each as global internal standards (GIS) [17]. All individual samples and the GIS standards were then dried using a speed vacuum. Six TMT batches were balanced for diagnosis, age, and sex using ARTS (automated randomization of multiple traits for study design) [18]. Using an 18-plex Tandem Mass Tag (TMT-pro) kit (ThermoFisher, Lot# UK297033 and WI336758), 17 channels were allocated for CSF samples with the remaining channel (126) containing a GIS pool, as described in [14].

### High-pH peptide fractionation

Dried samples were re-suspended in high pH loading buffer (0.07% vol/vol NH4OH, 0.045% vol/vol FA, 2% vol/vol ACN) and loaded onto a Waters BEH column (2.1 mm × 150 mm with 1.7 µm particles). A Vanquish UPLC system (ThermoFisher Scientific) was used to carry out the fractionation. Solvent A consisted of 0.0175% (vol/vol) NH4OH, 0.01125% (vol/vol) FA, and 2% (vol/vol) ACN; solvent B consisted of 0.0175% (vol/vol) NH_4_OH, 0.01125% (vol/vol) FA, and 90% (vol/vol) ACN. The sample elution was performed over a 25 min gradient with a flow rate of 0.6 mL/min with a gradient from 0 to 50% solvent B. A total of 96 individual equal volume fractions were collected across the gradient. Fractions were concatenated to 96 fractions and dried to completeness using vacuum centrifugation.

### Mass-spectrometry analysis and data acquisition for TMT

All samples (~ 1 µg for each fraction) were loaded and eluted by an Ultimate 3000 RSLCnano (Thermo Scientific) with an in-house packed 20 cm, 150 μm i.d. capillary column with 1.7 μm CSH (Waters) over a 22 min gradient. Mass spectrometry was performed with a high-field asymmetric waveform ion mobility spectrometry (FAIMS) Pro front-end equipped Orbitrap Eclipse (Thermo) in positive ion mode using data-dependent acquisition with 1.5 s top speed cycles for each FAIMS compensative voltage. Each cycle consisted of one full MS scan followed by as many MS/MS events that could fit within the given 1 s cycle time limit. MS scans were collected at a resolution of 120,000 (410–1600 m/z range, 4 × 10^5 AGC, 50 ms maximum ion injection time, FAIMS compensative voltage of − 45 and − 65). Only precursors with charge states between 2 + and 6 + were selected for MS/MS. All higher energy collision-induced dissociation (HCD) MS/MS spectra were acquired at a resolution of 30,000 (0.7 m/z isolation width, 35% collision energy, 1 × 10^5 AGC target, 54 ms maximum ion time, turboTMT on). Dynamic exclusion was set to exclude previously sequenced peaks for 20 s within a 10-ppm isolation window.

### Database search and protein quantification

Database searches and protein quantification was performed on 576 RAW files (96 RAW files/fractions per batch) using FragPipe (version 19.1). The FragPipe pipeline relies on MSFragger (version 3.7) [19, 20] for peptide identification, MSBooster [21], and Percolator [22] for FDR filtering and downstream processing. MS/MS spectra were searched against all canonical Human proteins downloaded from Uniprot (20,402; accessed 02/11/2019), as well as common contaminants (51 total), and all reverse sequences (20,453). The workflow we used in FragPipe followed default TMT- 18 plex (i.e., TMTpro) parameters. Briefly, precursor mass tolerance was − 20 to 20 ppm, fragment mass tolerance of 20 ppm, mass calibration and parameter optimization were selected, and isotope error was set to − 1/0/1/2/3. Enzyme specificity was set to strict-trypsin with up to two missing trypsin cleavages allowed. Cleavage was set to semi-tryptic, peptide length was allowed to range from 7 to 35 and peptide mass from 200 to 5,000 Da. Variable modifications that were allowed in our search included: oxidation on methionine, N-terminal acetylation on protein and peptide, TMT labeling reagent modifications on serine, threonine, and histidine with a maximum of 3 variable modifications per peptide [23]. The false discovery rate (FDR) threshold was set to 1%. A total of 49,762 peptides which mapped to 2,568 proteins were detected. After filtering out proteins that were absent in 50% or more of specimens 23,743 peptides and 2,105 proteins were retained (Supplemental Tables 3 and 4).

### DIA-MS Proteomics

An expanded cohort incorporating four centers was included for the purpose of confirming findings from the original dataset and, as a secondary objective, to validate the initial analysis across an additional, unbiased data-independent acquisition mass spectrometry (DIA-MS) approach. All samples (20 µL per sample) were digested (5 µg of peptides) as described above. Samples (1.6 µL) of digested CSF equivalent were then loaded and eluted by a Bruker timsTOF HT coupled with an Evosep One LC system. Data was acquired at 30 SPD with Performance Column (8 cm × 150 um × 1.5 um, part number EV1109). DIA with narrow mobility window 0.85–1.35 V s cm − 2 (volts x seconds/square cm). Database searches and protein quantification was performed on 302 RAW files using Spectronaut (version 18.1; [24]) and default, fully tryptic parameters (Supplemental Table 5). The database used for this search was identical to the TMT-MS search.

### Bioinformatics processing and statistical analysis

We employed a Tunable Approach for Median Polish of Ratio (TAMPOR) on mode 3 for TMT-MS and mode 4 for DIA-MS [25] and removal of peptides or proteins absent in 50% of cases or greater, as previously published [14, 26, 27]. Multidimensional scaling was used to visualize distribution of post-TAMPOR data as a quality control (Supplemental Fig. 1). To ensure the reliability of our data, we initiated the analysis by identifying and removing potential outliers, although none were present. We then performed parallelized [28] ordinary, nonparametric, bootstrapping regression to remove variation due to age, sex, and batch effect on TMT-MS and age and sex on DIA-MS, which lacked batches, using an established pipeline [7, 27]. Fast parallel one-way ANOVA with Benjamini–Hochberg correction for multiple comparisons was conducted within each disease group using an in-house script (https://github.com/edammer/parANOVA) to identify peptides and proteins that were differentially expressed (Supplemental Tables 6, 7 and 8). Differential abundance is presented as volcano plots, which were generated with the ggplot2 package [29].

### Protein network analysis

Weighted Gene Co-expression Network Analysis (WGCNA) [30] was used to construct modules of co-expressing proteins as previously published [7, 12]. Briefly, the blockwiseModules function from the WGCNA package in R was utilized with the following parameters for Emory ALS CSF samples: soft threshold power beta = 4, deepSplit = 4, minimum module size = 15, merge cut height = 0.07, and a signed network with partitioning around medoids (mapping a distance matrix to k clusters, where K is data-adaptively selected; Supplemental Table 9) [31]. Module correlation to disease type was evaluated with biweight midcorrelation (BiCor) analysis by separating each disease control combination. Fisher’s exact test (FET) was performed for each module’s members against the merged human brain cell type marker list to determine cell type enrichment using an in-house script (https://github.com/edammer/cellTypeFET). Similarly, to determine module gene ontology (GO) a FET was performed for each module member against the Bader Lab’s GMT formatted ontology lists from February 8, 2023 [32] (https://github.com/edammer/GOparallel; Supplemental Table 10). One way ANOVA was conducted across all disease type groups for each module eigenprotein.

Assessment of module preservation between the Emory and the multi-center datasets was done using the modulePreservation function of WGCNA with 500 permutations [7, 27, 33]. Synthetic module eigenproteins (MEs) were calculated using the top 20 percent of hubs ranked by kME_intramodule_ in the single center dataset used to build the template network, and a minimum of 4 such hubs found in the expanded dataset (Supplemental Table 11). The first principal component of the selected proteins in the mapped dataset was calculated as the synthetic ME in that data using the moduleEigengenes function of WGCNA R package as in [7, 27, 33].

In order to assess overlap of proteins by symptom status (i.e. sALS versus control, SOD1 versus control, C9orf72 versus control, etc.) we scattered comparisons and restricted proteins to those significantly different in both comparisons (*p* < 0.05). Effect sizes were scattered for each symptom status and the bicor And *P* value function of the WGCNA R package was used to assess correlation and significance. The most differentially abundant (top 12) proteins between ALS and control as well as symptomatic versus asymptomatic were determined using ANOVA. These proteins were used in each case to perform a principal component analysis and hierarchical clustering. In both cases these analyses separated the samples by symptom status. Finally, we connected all differentially abundant proteins across ALS versus control and symptomatic versus asymptomatic to the module network structure.

## Results

### Experimental workflow identifies differentially expressed proteins across ALS subtypes

**Fig. 1:**
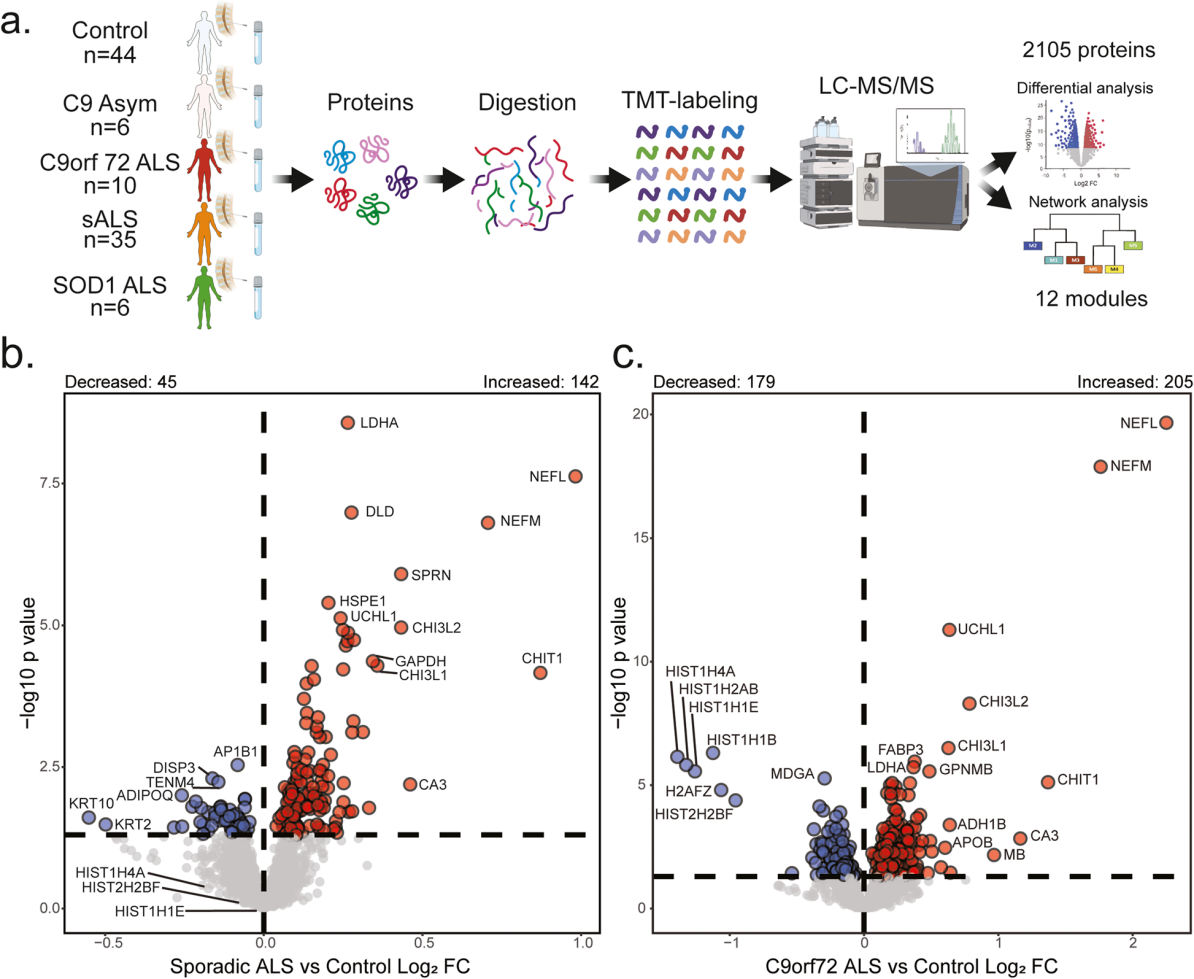
Experimental workflow with differential expression of ALS versus control CSF proteomes using TMT-MS. **a**. Schematic of experimental workflow to examine proteomic differences in cerebrospinal fluid (CSF) between subjects with ALS and controls. **b**-**c**. Volcano plots showing differential abundance profiles comparing control CSF (*n* = 44) to that from sALS (*n* = 35) and C9orf72 ALS (*n* = 10). Log_2_ fold change (x-axis) and one-way ANOVA with Benjamini–Hochberg corrected by disease -log10 *p*-values (y-axis). Note the commonly increased CSF proteins in patients with ALS such as NEFL, NEFM, and CHIT1. Proteins significantly (*p* < 0.05) increased in abundance are depicted in red, significantly decreased in blue, and neither in grey

Using the single center Emory CSF cohort, we compared CSF proteomes from sALS (*n* = 35), C9orf72 ALS (*n* = 10), C9orf72 asymptomatic carriers (*n* = 6), SOD1 ALS (*n* = 6), and healthy controls (*n* = 44) with the goals of discovering differences between ALS and control, and protein signatures that may differentiate the ALS subtypes (Fig. 1a). TMT-MS proteomic analysis represented 2,105 proteins with more than 50 percent of all samples having quantification. Protein abundance was adjusted for batch, age, and sex [12, 14]. As expected, protein levels of neurofilaments (NEFM and NEFL) were increased in both sporadic and genetic ALS subtypes compared to controls, consistent with neurodegeneration [34–36]. Furthermore, we observed an increase in chitinases, CHIT1 and CHI3L1, linked to inflammation and previously shown to increase in ALS [37, 38].

Comparison of the CSF proteome between sporadic ALS and controls, as well as C9orf72 ALS and controls, revealed many shared differentially abundant proteins (DAPs) between the ALS subtypes. (Figs. 1b and 1c; Supplemental Fig. 2; Supplemental Table 6). However, proteins with relative increased abundance in C9orf72 ALS compared to sALS (such as NEFL, NEFM, IGHM, and ADH1B) included those with axonal regeneration ontology as well as proteins with metabolic pathways associated with L-ascorbic acid, cellular ketone, and cellular aldehyde. Proteins including HIST1H2 AB, HIST1H1B, HIST1H1E, HIST1H4 A, H2 AFZ, and HIST2H2BF as well as those related to chemical synaptic transmission and assembly of postsynaptic elements in C9orf72 ALS CSF were significantly decreased in abundance relative to sALS.

### Network analysis of the ALS CSF proteome reveals modules related to brain pathways, brain cell-types and genetic background

We utilized Weighted Gene Co-expression Network Analysis (WGCNA) [30] to identify modules or ‘communities’ of proteins that are highly correlated across CSF samples. These modules in CSF reflect various biological functions linked to brain cell types and ontologies [13, 14]. Using the first principal component of all proteins in a module, or the module eigenprotein, we can relate the abundance of each module to disease phenotypes with greatly reduced reliance on multiple testing.

**Fig. 2:**
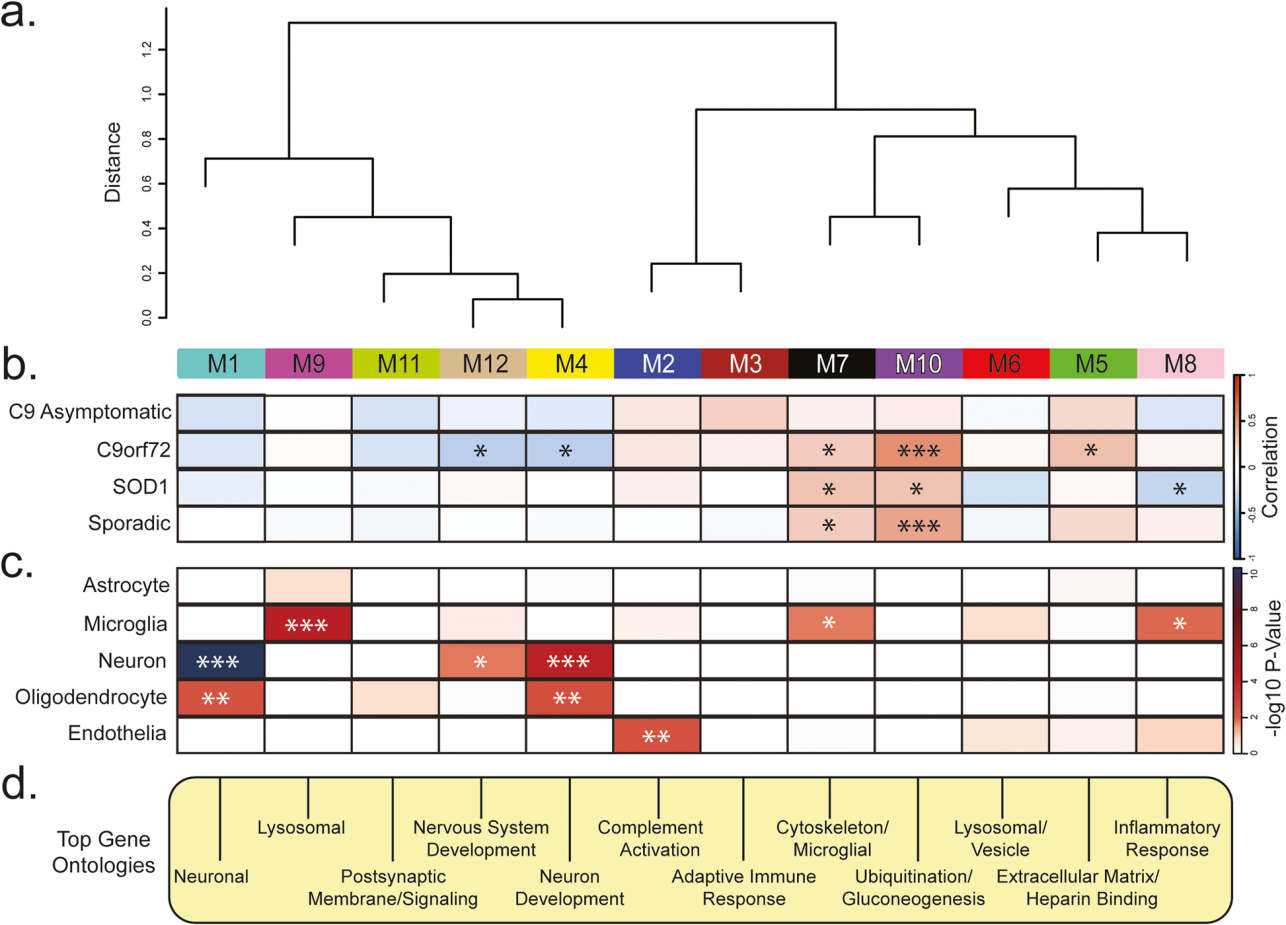
CSF Network modules associate with brain cell-types and ALS disease subtypes. **a**. Cluster dendrogram indicates similarity of WGCNA network modules based on correlation of eigenproteins (i.e., first principal component). **b**. Relationship between ALS disease subgroup (sporadic, SOD1, Asymptomatic C9orf72 and symptomatic C9orf72) with individual protein modules was evaluated by cross referencing trait values and module proteins using a Biweight midcorrelation (BiCor) analysis. Significance as determined by BiCor are denoted by overlain asterisks; *p < 0.05, **p < 0.01, ***p < 0.001. Note the relatedness of modules 7 and 10 as well as the overlap in significance between these modules by disease subtype compared to control group. **c.** Cell-type enrichment was characterized by comparing module proteins with a list of proteins known to be enriched in astrocytes, microglia, neurons, oligodendrocytes, and endothelia; respectively (see methods). Significance levels determined by one-tailed Fisher’s exact test are denoted by overlain asterisks; *p < 0.05, **p < 0.01, ***p < 0.001. **d**. Top gene ontology (GO) terms were selected from significant GO annotations (Supplemental Table 10)

Here, our network modules ranged from 476 (M1) to 29 (M12) member proteins with 81% of proteins mapping to a module. The 12 network modules were generally divided into three branches of relatedness, allowing us to infer which modules were most similar to each other (Fig. 2a). We compared correlation between specific disease groups and network module co-expression to determine that five modules were associated with C9orf72, three with SOD1, and two with sALS, with overlap in module identity (Fig. 2b). We also evaluated whether proteins associated with specific brain cell types were enriched in certain modules [14], potentially indicating relative changes in CNS cell type abundance or activity caused by disease. Three modules showed significant enrichment for neuronal markers (M1, M4, and M12) and microglial markers (M7, M8, and M9). Additionally, two modules were enriched for oligodendrocyte markers (M1 and M4), and one module was enriched for endothelial markers (M2; Fig. 2c). Modules showing a high degree of correlation did not necessarily have more overlap with protein markers of cell type. Specifically, M1 and M4 were significantly enriched with neuronal and oligodendrocyte associated proteins but were more closely correlated to modules nine and 12; respectively (Fig. 2c). The three largest modules corresponded to M1- Neuronal, M2-Complement activation, enriched with markers specific to endothelia, and M3-Adaptive immune response ontologies (Fig. 2d). Smaller modules were associated with M4-Neuron development, M5-Extracellular matrix/Heparin binding, M6-Lysosomal/Vesicle, M7-Cytoskeleton/Microglial, M8-Inflammatory Response, M9-Lysosome, M10-Ubiquitination/Gluconeogenesis, M11-Postsynaptic membrane/Signaling, and M12-Nervous system development (Fig. 2d; Supplemental Table 10).

**Fig. 3:**
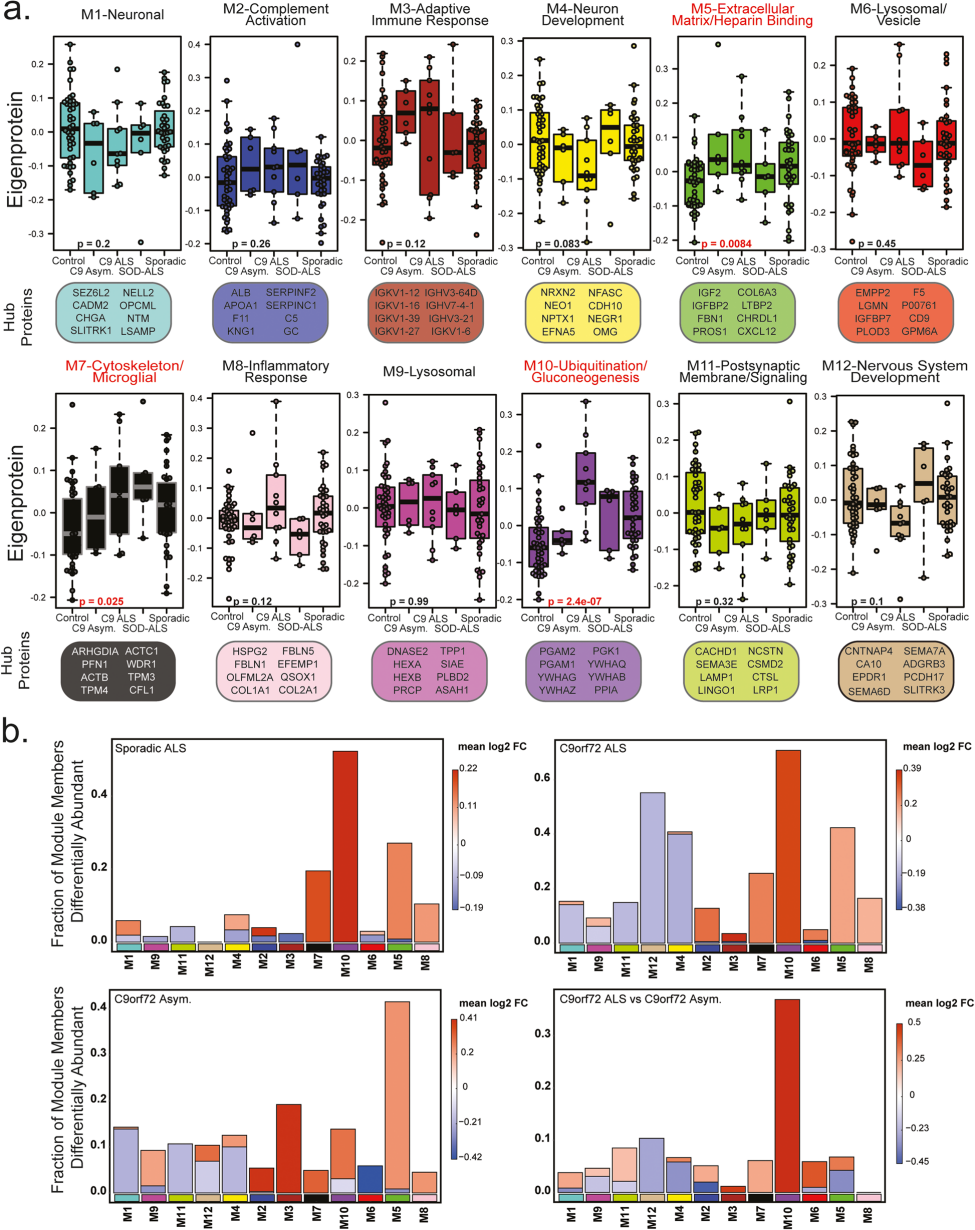
CSF Network Modules representing diverse biology vary across ALS subgroups. **a**. Eigenproteins for each of the CSF proteome modules (*n* = 12) were compared across control and ALS disease sub-type by one-way ANOVA. Hub proteins and GO terms for each module are highlighted. **b**. Differentially abundant proteins from each subgroup compared to controls for C9 ALS, Sporadic ALS and C9 Asymptomatic were mapped by module. Symptomatic vs asymptomatic C9orf72 differences were also included. The height of the bars represents the fraction of module member proteins that were differentially abundant. The bars are color coded by heatmap for average log_2_ difference in abundance, where red represents an increase in abundance, and blue represents a decrease in abundance

Modules associated with M5-Extracellular matrix/Heparin binding (*p* = 0.0084), M7-Cytoskeleton/Microglia (*p* = 0.025), and M10-Ubiquitination/Gluconeogenesis (*p* = 2.4e- 07) varied significantly among control and ALS groups (Fig. 3a). To reinforce these findings, most of the increased DAPs in ALS cases irrespective of genetic cause mapped to M5, M7 and M10, whereas decreased DAPs in ALS were distributed in M4, M11, and M12 (Fig. 3b). DAPs in C9orf72 patients increased in abundance also contributed to these modules, with lowered abundance proteins belonging to modules associated with M4-Neuron development and M12-Nervous system development. Proteins increased in abundance in asymptomatic C9orf72 cases were more commonly clustered into the module associated with M5-Extracellular matrix/Heparin binding and proteins that were significantly increased in symptomatic C9orf72 relative to sporadic cases, including neurofilaments, chitinases, and deubiquitinases belonging to the module associated with M10-Ubiquitination/Gluconeogenesis (Fig. 3b). Module overarching ontologies and correlation helped identify broad protein functions more closely associated with C9orf72 (M5-Extracellular matrix/Heparin binding) as well as the degree to which these functions overlapped (i.e. all ALS subgroups correlated to M7-Cytoskeleton/Microglial and M10-Ubiquitination/Gluconeogenesis). Overall, network analysis effectively organizes the CSF proteome into protein modules that are strongly linked to hallmark ALS and neurodegenerative biomarkers.

### Validation of CSF protein abundance changes in an expanded multicenter cohort

**Fig. 4:**
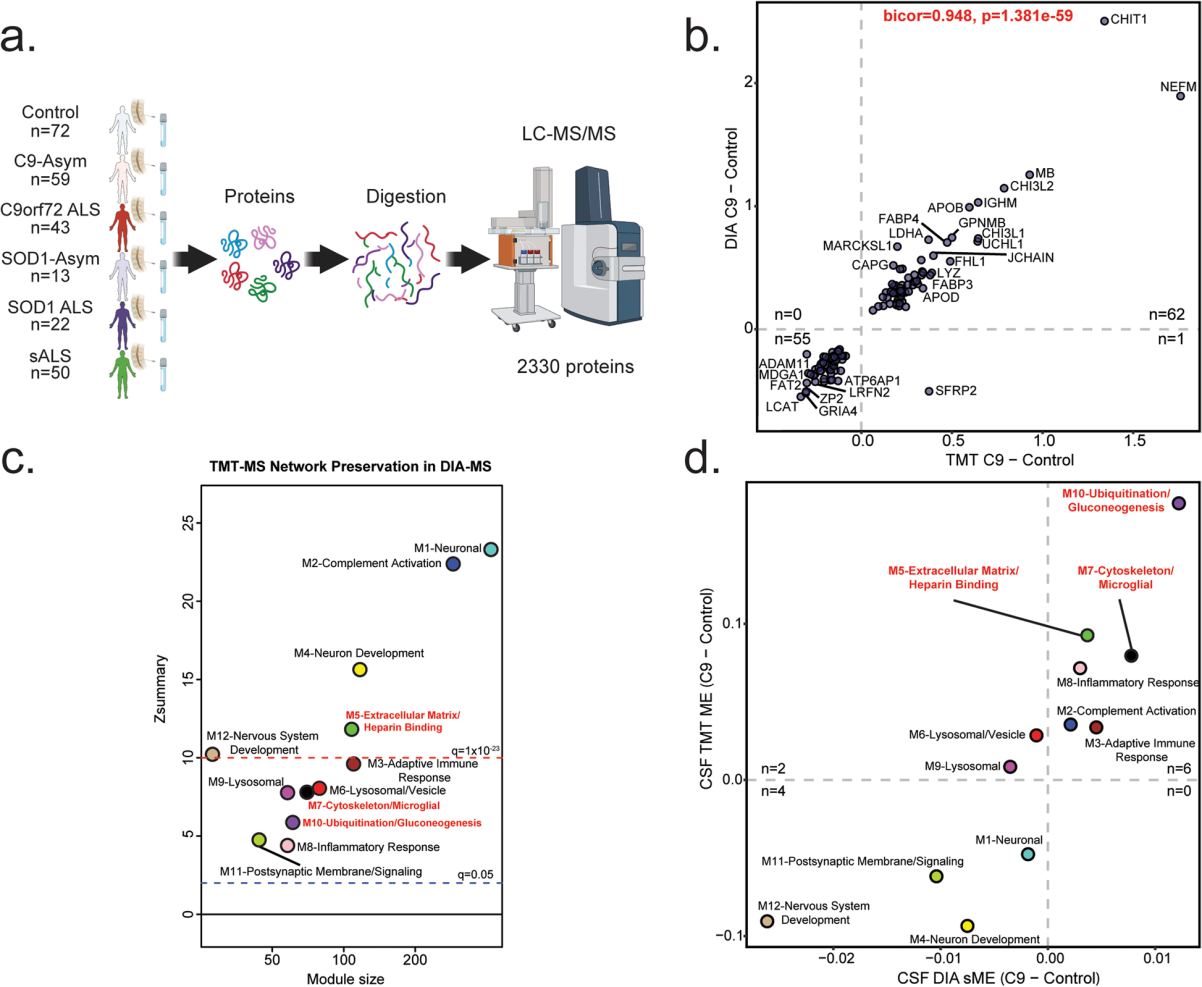
ALS CSF protein network changes are preserved in a larger multicenter cohort. **a**. Schematic of experimental workflow to quantitatively evaluate an additional CSF proteome generated DIA-MS (multicenter dataset), indicating sample size and number of proteins quantified. Note the addition of an asymptomatic SOD1-mutation group. **b**. Scatter plot showing the overlap in effect size in the subset of individuals included in both TMT-MS and DIA-MS (*N* = 50, C9orf72 = 10, Control = 40). Proteins that varied significantly in both sets were compared using BiCor and associated Student correlation *p*-value. The number of proteins in each quadrant is denoted by “n”. **c**. Module preservation of TMT-MS (original dataset) and DIA-MS (expanded dataset). Number of proteins in each module (x-axis) is compared across Zsummary and overall measurement of preservation, (y-axis). The red line at Zsummary = 10 (*q* = 1 × 10^–23^) indicates TMT-MS modules are highly preserved in the replication proteome. The blue line at Zsummary = 2 (*q* = 0.05) indicates TMT-MS modules are preserved in the DIA-MS dataset. **d**. Scatter plot correlating module eigenproteins for C9orf72 vs. control for TMT-MS (y-axis) and DIA-MS (x-axis). 10 of 12 network modules correlate between the two methods. Synthetic eigenproteins were constructed for DIA-MS dataset and measured by disease type in TMT-MS datasets. A minimum of four proteins from the top 20% of module membership by kME (correlation to module eigenprotein) were used to assess synthetic eigenprotein value (y-axis) and compared across disease type (x-axis) (Supplemental Table 11)

To assess the consistency of our findings from the Emory CSF cohort, we analyzed a separate dataset of CSF samples using unbiased label free data-independent acquisition mass spectrometry (DIA-MS). This analysis was conducted across four independent centers, adding 158 individuals unique to the study. We compared the Emory CSF proteome to the expanded multicenter proteome (Fig. 4a) at both the individual protein and network module level. Of the 2,105 and 2,330 proteins identified by TMT-MS from the Emory and DIA-MS from the expanded cohort, respectively, 81.8 and 73.9% of those proteins were shared. The Emory TMT-MS dataset included 384 proteins not found in the expanded proteome whereas the DIA-MS dataset included 609 proteins not found in Emory. However, comparing the shared DAPs between common individuals in the C9orf72 ALS vs. control comparison of the two datasets we found a high degree of correlation (bicor = 0.948, *p* = 1.381e- 59). For this comparison, 117 DAPs coincided in direction of change, while only 1 DAP protein differed (Fig. 4b). Note, that neurofilament proteins are poorly characterized in DIA-MS as compared to TMT-MS [39], and so the effect size of neurofilament in the DIA-MS analysis is blunted.

To assess module preservation across the Emory and Multicenter ALS cohorts, we constructed a protein co-expression adjacency matrix for the DIA-MS ALS cohort and evaluated the preservation of TMT-derived Emory network modules in the multicenter dataset. Notably, modules M1-Neuron, M2-Complement activation, M4-Neuron development, M5-Extracellular matrix/Heparin binding, and M12-Nervous system development had Z_summary_ values exceeding 10 (*q* = 1 × 10^–23^), indicating strong preservation. Modules M3-Adaptive immune response, M6-Lysosomal/Vesicle, M7-Cytoskeleton/Microglia, M8-Inflammatory response, M9-Lysosome, M10-Ubiquitination/Gluconeogenesis, and M11-Postsynaptic membrane/signaling showed Z_summary_ values between 2 (*q* = 0.05) and 10, reflecting moderate to high preservation (Fig. 4c).

**Fig. 5:**
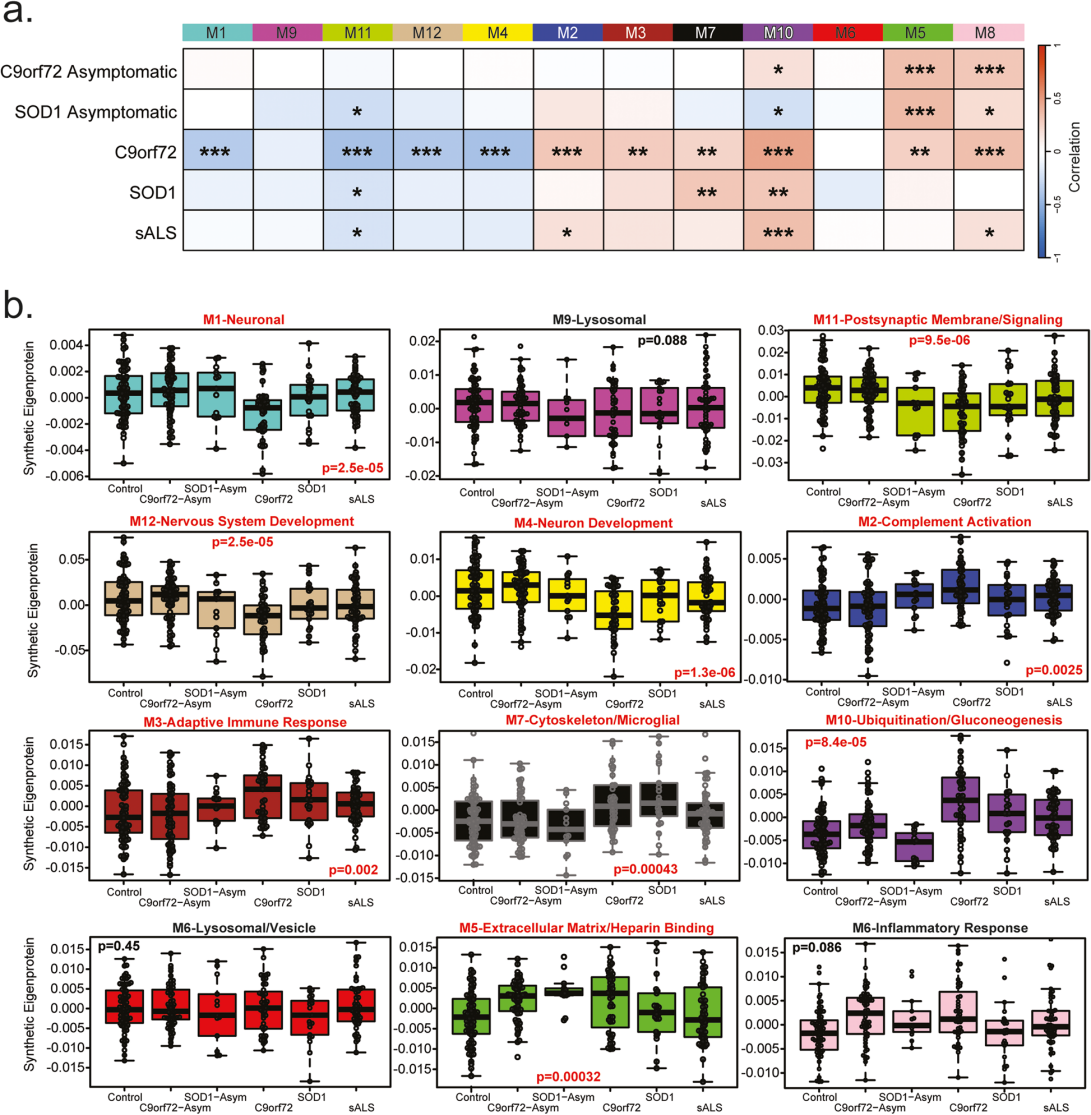
Synthetic eigenproteins indicate variation between different forms of ALS, relative to controls. **a**. Relationship between symptom status (asymptomatic C9orf72, asymptomatic SOD1, C9orf72 ALS, SOD1 ALS, sporadic ALS) modules was evaluated by cross referenced trait values with module proteins using a Biweight midcorrelation (BiCor) analysis. Significance as determined by BiCor are denoted by overlain asterisks; *p < 0.05, **p < 0.01, ***p < 0.001. **b**. Synthetic eigenproteins for each of the CSF proteome modules (*n* = 12) were compared across control and ALS disease sub-type by one-way ANOVA

To evaluate module directionality across disease subgroups between the two cohorts, we compared module eigenproteins from the Emory cohort with the same"synthetic"eigenproteins from the multicenter cohort generated as described [27]. Ten of the 12 modules showed concordant changes, while M6-Lysosomal/Vesicle and M9-Lysosome exhibited a slight increase in the Emory cohort but a decrease in the expanded dataset (Fig. 4d), yet these modules were not different by disease in either the Emory or multi-center cohort. Importantly, all synthetic module eigenproteins corresponding to differentially abundant modules in the Emory dataset (M5-Extracellular matrix/Heparin binding, M7-Cytoskeleton/Microglial, and M10-Ubiquitination/Gluconeogenesis) were also differentially abundant in the expanded multicenter dataset. Additionally, six other modules showed differences across symptom status, likely due to the increased statistical power of the larger multi-center cohort (Fig. 5).

The direction of the modules across ALS subtypes stratified by the presence or absence of C9orf72 or SOD1 mutation in the DIA-MS was highly consistent to that of the TMT-MS datasets (Fig. 5a). All ALS cases were correlated to two modules, one positive (M10) and one negative (M11). While genetic subtypes (SOD1 and C9orf72) were also positively correlated to an additional module (M7). The directionally of module change was mainly consistent between asymptomatic and symptomatic ALS cases. Interestingly, M10, the module most increased across all symptomatic ALS cases was decreased in asymptomatic SOD1 carriers, but increased in asymptomatic C9orf72 carriers, suggesting the trajectory of these proteins changes differs by genetic subtype (Fig. 5b; Supplemental Fig. 3). Thus, the validation of CSF protein abundance changes in an expanded multicenter ALS cohort demonstrates strong reproducibility of findings from the Emory dataset. Despite differences in mass spectrometry platforms (TMT-MS vs. DIA-MS) and sample sources (single-site vs. multi-site), there was substantial overlap in identified proteins, a high correlation in differentially abundant proteins (DAPs), and strong preservation of protein co-expression network modules.

### Differentially Abundant Proteins in symptomatic and asymptomatic gene mutation carriers

The expanded multi-center cohort provided greater statistical power to compare different ALS subtypes with controls and asymptomatic gene carriers. These comparisons identified DAPs and highlighted similarities and differences across ALS subtypes. In the comparison of C9orf72 asymptomatic carriers to controls, 383 DAPs were identified, with 205 increased and 178 decreased (Supplemental Fig. 3a). The histone protein HIST1H4 A was significantly decreased while LDHA, SNCB, CXCL12, and FABP5 were among those increased in abundance. DAPs in asymptomatic C9 carriers that showed an increase in abundance, predominantly clustering in M5 (Extracellular Matrix/Heparin Binding) and M8 (Inflammatory Response). In contrast, DAPs that decreased in abundance were more evenly distributed but were primarily associated with functionally related modules, including M1 (Neuronal), M4 (Neuron Development), M11 (Postsynaptic Membrane/Signaling), and M12 (Nervous System Development). (Supplemental Fig. 3a). In asymptomatic SOD1 relative to controls, 394 DAPs were present (183 increased and 211 decreased; Supplemental Fig. 3b). As in asymptomatic C9orf72 carriers, the histone protein HIST1H4 A was reduced, along with the addition of HIST1H2 AB. Notably, both SOD1 and SOD2 were also reduced, mirroring the changes observed in the asymptomatic SOD1 group compared to controls. DAPs increased in abundance were most consistently nested in M5, while decreased abundance DAPs were associated disproportionately with M11, M9 (Lysosomal), and M12 (Supplemental Fig. 3b). A direct comparison showed more DAPs were unique to each asymptomatic condition than were shared (Supplemental Fig. 3c).

**Fig. 6:**
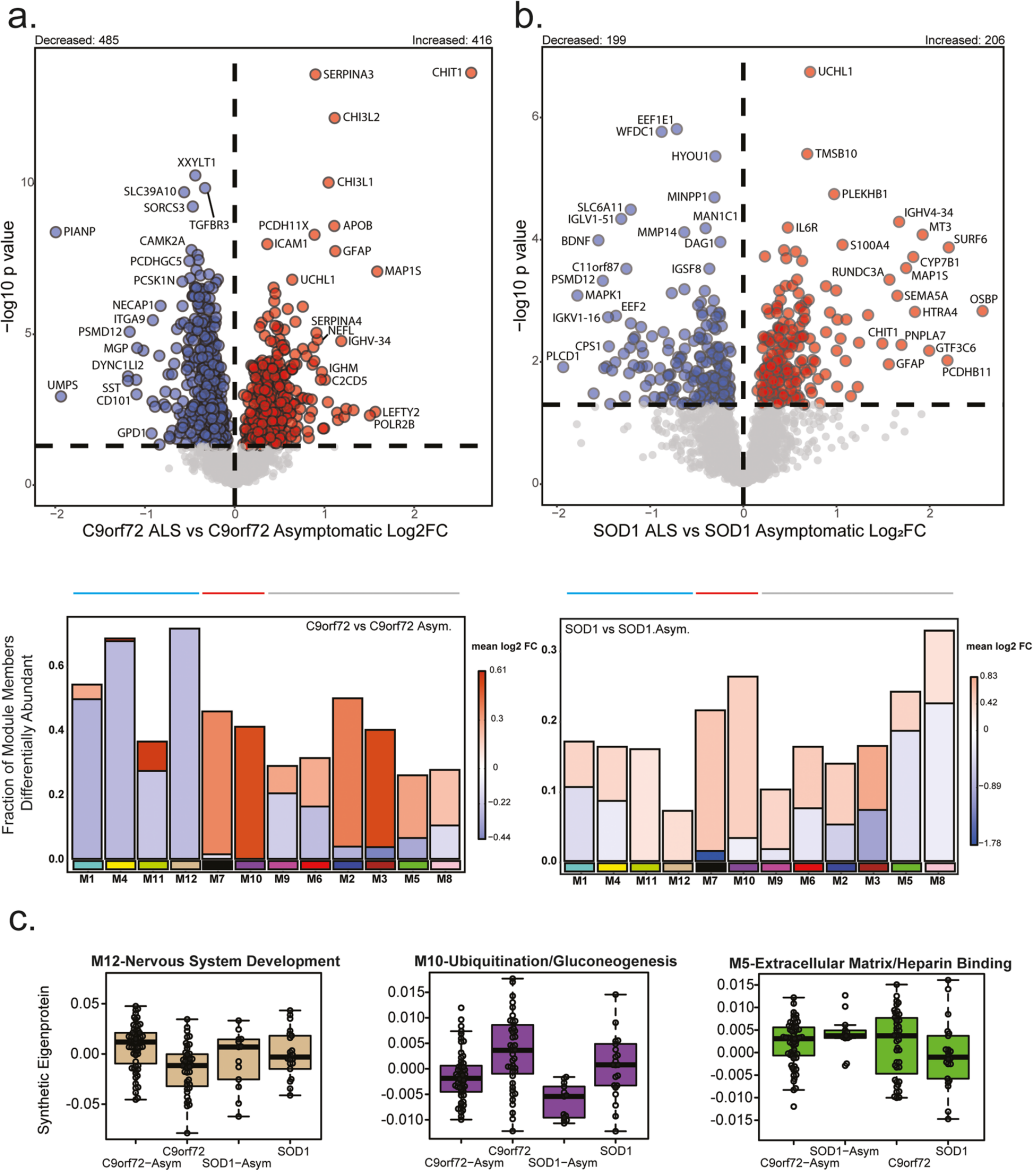
Asymptomatic individuals were distinct from symptomatic individuals with these differences indicating novel biology. **a**. Volcano plot showing differential abundance profiles comparing asymptomatic C9orf72 carriers (*n* = 59) and symptomatic C9orf72 carriers (*n* = 43). Differentially abundant proteins were mapped by module. The height of the bars represents the fraction of module member proteins that were differentially abundant. The bars are color coded by heatmap for average log_2_ difference in abundance, where red represents an increase in abundance, and blue represents a decrease in abundance. **b** Asymptomatic SOD1 (*n* = 13) compared to symptomatic SOD1 carriers (*n* = 22). Differentially abundant proteins were also mapped by module. Log_2_ fold change (x-axis) and one-way ANOVA with Benjamini–Hochberg corrected by disease -log10 *p*-values (y-axis). Proteins significantly (*p* < 0.05) increased in abundance are depicted in red, significantly decreased in blue, and neither in grey. **c**. Representative modules of groups outlined above stacked barplots are depicted restricted to subtypes depicted in volcano plots

To identify shared and distinct differences between asymptomatic and symptomatic disease that may be linked to disease progression across SOD1 and C9orf72 ALS subtypes, we directly compared asymptomatic carriers to symptomatic carriers within each subgroup. In individuals with the C9orf72 mutation, 901 DAPs were detected (416 increased, 485 decreased; Fig. 6a). Notably, CHIT1, CHI3L1, CHI3L2, UCHL1, GFAP, and NEFL were elevated, likely reflecting neuroinflammation and degeneration associated with disease onset. DAPs increased in abundance were strongly associated with related modules M7 (Cytoskeleton/Microglial) and M10 (Ubiquitination/Gluconeogenesis) as well as M2 (Complement Activation) and M3 (Adaptive Immune Response). Decreased DAPs were in M1, M4, M11, and M12, similar to asymptomatic C9orf72 versus control, but more consistently (Fig. 6a).

For SOD1 mutation carriers, 405 DAPs were identified between asymptomatic and symptomatic individuals (206 increased, 199 decreased; Fig. 6b). Similar to C9orf72 carriers, UCHL1, CHIT1, and GFAP as were most proteins in M10 and M7 (Cytoskeleton/Microglial). DAPs mapping to M7 and M10 were consistently increased in both C9orf72 and SOD1 ALS cases compared to genotype specific asymptomatic controls indicating that these are share pathways and biomarkers across these familial forms of ALS. In contrast, while modules M1, M4, M11, and M12 were predominantly decreased in C9orf72 ALS, proteins mapping to these modules in SOD1 ALS showed a moderate increase. This suggests a more significant reduction of synaptic biomarkers in C9orf72 ALS compared to SOD1 ALS, which may be due to distinct differences in underlying pathology of these diseases. Notably, M5-Extracellular matrix/Heparin binding and M8 (Inflammatory response) had a higher proportion of decreased DAPs in SOD1 compared to C9orf72 highlighting other divergent modules between the genetic subtypes (Fig. 6).

The inclusion of additional SOD1 cases in the expanded cohort enabled a more robust analysis of DAPs in symptomatic and asymptomatic SOD1 mutation carriers, as well as comparisons across different SOD1 mutations. In the comparison of SOD1 ALS vs. controls, 456 DAPs were identified, with 186 proteins increased and 270 decreased (Supplemental Fig. 4a). DAPs that were increased were associated with M7 and M10, which is shared with symptomatic relative to asymptomatic SOD1. DAPs that were mixed (including increased and decreased) were distributed among M2, M3, M5, and M8. While M1, M4, M11, M12, M9, and M6 (Lysosomal/Vesicle) contained largely decreased abundance proteins. M11, in particular, was consistently populated with decreased DAPs.

Notably, SOD1 protein levels were reduced in both symptomatic SOD1 ALS and asymptomatic SOD1 carriers compared to controls (Supplemental Fig. 3b; Supplemental Fig. 4a). Further analysis revealed that this reduction was primarily driven by carriers of the A5 V and A5 T mutations (Supplemental Fig. 4b), which are known to be among the most clinically aggressive SOD1 mutations [40]. Several mutations in the cohort were captured by peptides quantified in the dataset (Supplemental Fig. 4c), showing a consistent reduction in canonical SOD1 protein levels (Supplemental Fig. 4d). In these peptides, A5-mutants consistently exhibited the lowest SOD1 levels. In summary, the expanded multi-cohort CSF dataset improved statistical power, allowing for a detailed comparison of ALS subtypes with controls and asymptomatic gene carriers. Key findings included significant changes in differentially abundant proteins associated with neuroinflammation, degeneration, and metabolic pathways, with notable reductions in SOD1 protein levels in the most aggressive SOD1 mutations.

### Individual biomarkers can distinguish ALS versus control and symptomatic versus asymptomatic

To assess the ability of top differentially abundant proteins to stratify ALS subtypes, we applied principal component analysis (PCA) and hierarchical clustering to visualize relationships among individual CSF proteomes. Two methods were used to evaluate the robustness of these CSF panels in sample classification.

**Fig. 7:**
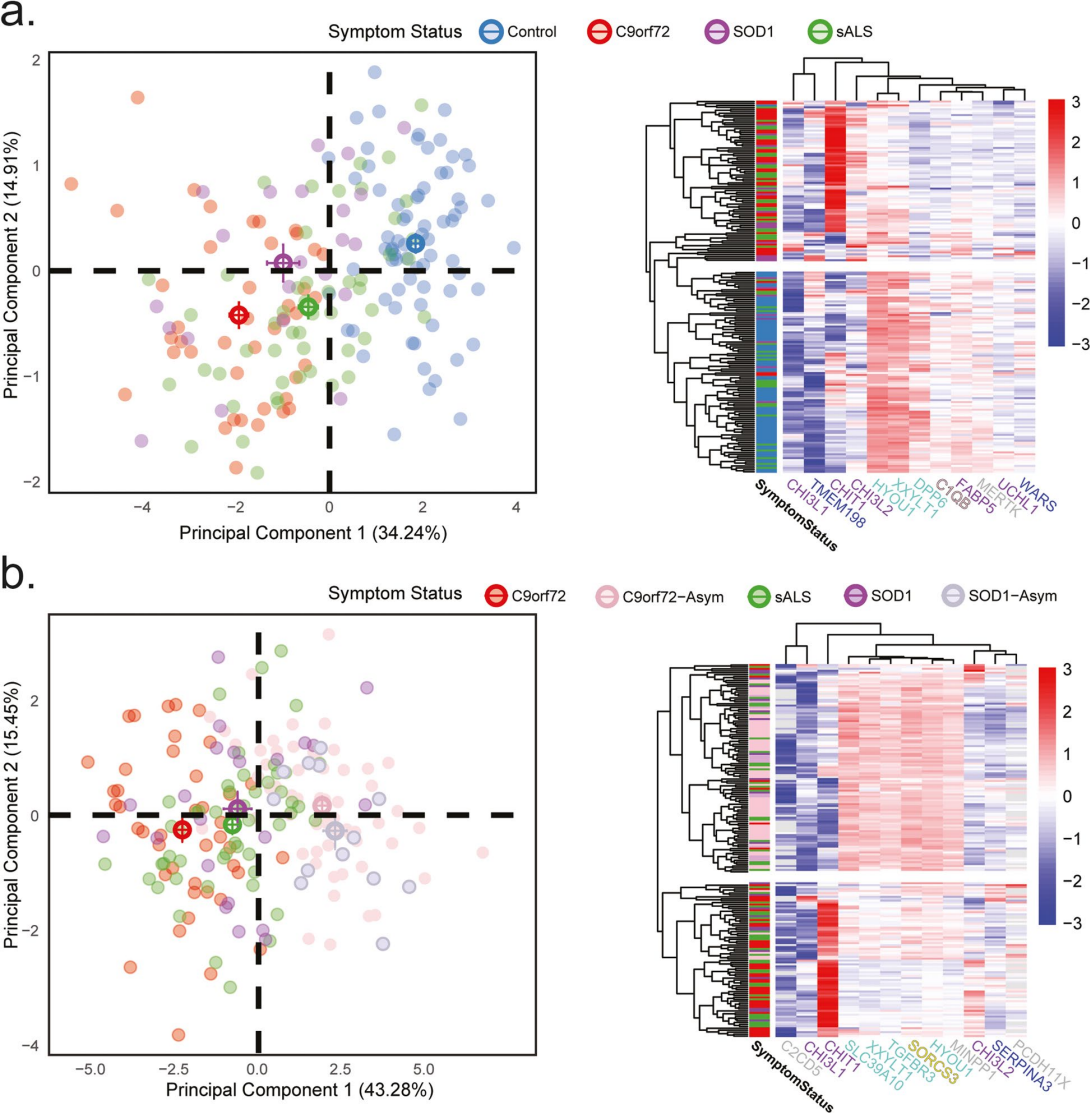
Comparisons of disease subtypes based on the most differentially expressed proteins. **a**. Principal component analysis of the top 12 differentially abundant proteins across all ALS and control individuals (*N* = 187). Individual points are transparent with centroids and standard error identified with crosses. Percent variation by principal component is presented on respective axes. Heatmap of normalized abundance for each of the 12 most variable proteins between all ALS and control, accompanied by hierarchical clustering of individuals and proteins. The heatmap separates control enriched (mostly blue) and ALS enriched (mostly red) classes. Proteins that varied most across ALS and control individuals are identified along the bottom of the heatmap. **b**. Principal component analysis depicting most variable proteins across all cases, both symptomatic and asymptomatic (*N* = 187). Individual points are transparent with centroids and standard error identified as crosses. Percent variation by principal component is presented on respective axes. Heatmap of normalized abundance for each of the 12 most variable proteins between symptomatic and asymptomatic individuals, accompanied by hierarchical clustering of individuals and proteins. The heatmap separates a symptomatic-enriched class (red and green) from an asymptomatic-enriched class (pink and pale purple). Proteins that varied most across symptomatic and asymptomatic individuals are identified along the bottom of the heatmap

Using the 12 most differentially abundant proteins (CHI3L1, TMEM198, CHIT1, CHI3L2, HYOU1, XXYLT1, DPP6, C1QB, FABP5, MERTK, UCHL1, and WARS), PCA effectively distinguished ALS from controls (Fig. 7a). While some overlap was observed among individual samples across ALS subtypes, ALS samples remained clearly separated from controls with sporadic ALS samples being the most heterogenous. Hierarchical clustering confirmed this trend, forming distinct ALS-enriched and control-enriched groups. While some of the most differentially abundant proteins were known ALS biomarkers (including CHIT1, CHI3L1, CHI3L2, FABP5, and UCHL1) [36, 37, 41], novel putative biomarkers were also identified (TMEM198, DPP6, C1QB, MERTK, WARS) as well as two ER related proteins (HYOU1 and XXYLT1).

Similarly, PCA using most the 12 most differentially abundant proteins (C2 CD5, CHI3L1, CHIT1, SLC39 A10, XXYLT1, TGFBR3, SORCS3, HYOU1, MINPP1, CHI3L2, SERPINA3, and PCDH11X) effectively distinguished asymptomatic from symptomatic ALS individuals (Fig. 7b). Moreover, unbiased hierarchical clustering identified distinct symptomatic- and asymptomatic-enriched groups. As with the analysis comparing ALS to controls novel putative markers were present including C2 CD5, SLC39 A10, TGFBR3, SORCS3, MINPP1, SERPINA2, and PCDH11X as well as the two ER related proteins in the prior analysis. Additionally, differentially abundant proteins in these comparisons showed overlapping module memberships, indicating shared biological pathways (Fig. 6). In summary, differentially abundant proteins stratify ALS subtypes, distinguishing cases from controls and symptomatic from asymptomatic individuals, revealing both known and novel biomarkers with shared biological pathways aligning with the CSF network.

## Discussion

In this study, we characterized the CSF proteome of subtypes of ALS defined by genetic status and clinical diagnosis to identify shared and unique biomarkers across each ALS subgroup. By examining coordinated protein co-expression across genetic and sporadic forms of ALS, we identified shared and group-specific changes linked to gene ontologies and cell-type functions in the proteome. We compared sALS, C9orf72 ALS and asymptomatic expansion mutation carriers, SOD1 ALS and asymptomatic SOD1 mutation carriers, and healthy controls to identify biomarkers that distinguish ALS from control, and asymptomatic from symptomatic gene carriers. Our analysis validated previously identified ALS associated CSF biomarkers [37, 41–43] but importantly, identified novel ALS biomarkers specific to genetic status as well as those that differentiate symptomatic from asymptomatic mutation carriers.

Our approach was to initially interrogate the Emory cohort given that the pre-analytical variables are best controlled when one site is considered. Using a uniformly sampled, single center Emory dataset, we recapitulated the expected result demonstrating that both C9orf72 and sALS proteomes vary from controls. While several of the most differentially variable proteins were shared across disease subtypes, distinct protein signatures were also observed for each subtype. Network modules captured diverse biology that further emphasize the differences in symptom status (i.e. asymptomatic vs. disease), and differences from control and from each other, particularly as it relates to M5-Extracellular matrix/Heparin binding, M7-Cytoskeleton/Microglia, and M10-Ubiquitination/Gluconeogenesis. Hub proteins for these modules represent novel biomarkers and potential interventional targets. The M5 and M10 network modules and their hub proteins similarly differentiate C9orf72 ALS from asymptomatic mutation carriers (see Fig. 3), suggesting these may reflect more generalized biomarkers of active disease.

An expanded, multicenter cohort was then assembled to validate the generalizability and robustness of the trends identified in the initial single-center dataset. At the individual protein and module level, a high degree of overlap in direction and magnitude was observed. Greater overlap in proteins increased in abundance relative to controls were observed in asymptomatic carriers—with the exception of some histone proteins. Similarly, in addition to known neurodegeneration biomarkers, numerous novel proteins were differentially abundant between symptomatic and asymptomatic carriers.

Using the most DAPs across ALS versus control, and symptomatic versus asymptomatic, we performed PCA analyses to plot all individuals in two dimensions. These two principal components separated all ALS groups from controls and even ALS groups from each other. This indicates that while the clinical presentations of ALS groups are largely indistinguishable, CSF proteomics suggests that pathobiology differs across those with sporadic versus genetic disease, as well as those with different disease-causing mutations. We also performed hierarchical clustering and characterized an ALS-enriched group and a control enriched group. Similarly, symptomatic and asymptomatic groups separated into distinct populations, with notable overlap between SOD1 ALS and sALS despite their differences in genetic underpinnings and pathological hallmarks. Across both analyses five proteins were shared including the chitinases (CHIT1, CHI3L1, and CHI3L2) and two ER related proteins (HYOU1 and XXYLT1). As recently reported [39] we saw a difference between UCHL1 in the ALS versus control comparison, and it is notable that a variant in DPP6 has been identified as a risk factor for ALS [44]. These two groups of proteins may prove useful as predictive biomarkers and in adding granularity of when individuals are responding to therapeutics.

An important result of this analysis is that both asymptomatic C9orf72 and SOD1 grouped together, separating proteomic signatures of asymptomatic gene carriers from symptomatic cases. Examples of differentially abundant proteins common to asymptomatic gene carriers are CXCL10, CXCL12, ELN, GAL3ST4, and PHGDH (all increased) and HIST1H4 A, PCDHB11, C2 CD5, GRHL2, and IL6R (all decreased). These similarities between asymptomatic gene carriers, with the notable exception of module 10, point to proteins that are potential biomarkers for transition from asymptomatic carrier to symptomatic disease. Although this will need to be tested in future longitudinal studies where pre and post symptomatic samples are available, it is crucial to identify biomarkers of conversion early to improve patient outcomes.

The characterization of asymptomatic carriers as well as the unprecedented number of SOD1-mutation carriers (*n* = 35, 22 SOD1 ALS, 13 asymptomatic carriers) differentiate this study from previous CSF biomarker studies [37, 41–43]. The increased number of SOD1 carriers in this expanded cohort allowed us to examine proteomic differences among the various SOD1 mutations. Interestingly, we found that, in general, people carrying SOD1 mutations had lower abundance of CSF SOD1 protein compared to control and to other ALS subsets. This was particularly evident for those with either the A5 T or A5 V variants, both symptomatic and asymptomatic, which are known to be associated with aggressive clinical phenotypes [40]. At the peptide level, these findings are largely recapitulated. A recent study [45] found that mutant SOD1 was 16-fold lower in concentration in CSF than wildtype SOD1 and that the turnover of mutant SOD1 was two times faster [45]. In conjunction with the finding that Tofersen lowered SOD1 by 30% [46], these results demonstrate the need to better understand how these concentrations are allocated amongst the myriad known SOD1 mutations and peptides.

## Conclusion

Our investigation presents the most extensive ALS-associated CSF proteome to date and identifies protein biomarkers that may distinguish the transition from asymptomatic to symptomatic phase, as well as proteomic differences separating genetic and sporadic forms that indicate differences in mechanisms of disease. We validated these biomarkers using two unbiased MS approaches across 259 individuals from four centers. Future analyses should utilize these differences to determine their applicability to diverse ALS cohorts, identify how these biomarkers develop longitudinally, and in additional tissue including spinal cord and motor cortex.

## Supplementary Information

Supplementary Material 1.

Supplementary Material 2. Supplemental Fig. 1. a. Multidimensional scaling was used on raw data that had been log-transformed to visualize multidimensional proteomic distribution in two-dimensional space. b. Post-TAMPOR mode 3 distribution of TMT-MS data removes batch-level effect present in raw data. c. Post-regression TMT-MS data indicate that distribution is not affected by methodological artifacts. d. Raw DIA-MS data processed with log-transformation demonstrate the difference in unprocessed DIA and TMT-MS. e. Post-TAMPOR mode 4 distribution of individual points show a reduction in any clustering that may be due to DIA-MS. f. Post-regression distribution is further removed of methodical influence. Panels a-c demonstrate distribution of TMT-MS proteome and panels and d-f demonstrate distribution of DIA-MS. Each datapoint represents an individual tissue sample with colors indicating, in TMT-MS, shared batch and, in DIA-MS, shared center of origin.
Supplemental Fig. 2. a. Shared and diverging differentially abundant proteins from single center TMT-MS quantification were compared between sporadic ALS and C9orf72 ALS. This scatterplot includes cases from the Emory single center dataset. Only proteins that were differentially abundant in both ALS subtypes are visualized. The number of proteins in each quadrant is denoted by “n”. b. Volcano plot showing differential abundance profiles comparing asymptomatic C9orf72 ALS (*n* = 10) and sporadic ALS (*n* = 35). Proteins that were significantly (p ≤ 0.05) down in disease (C9orf72 ALS, relative to control) are depicted in blue (*n* = 151), proteins that were significantly up are depicted in red (*n* = 139), and proteins that were neither significantly up nor down are grey.
Supplemental Fig. 3. a. Volcano plot showing differential abundance profiles comparing asymptomatic C9orf72 carriers (*n* = 59) and controls (*n* = 72). Differentially abundant proteins were mapped by module. The height of the bars represents the fraction of module member proteins that were differentially abundant. The bars are color coded by heatmap for average log_2_ difference in abundance, where red represents an increase in abundance, and blue represents a decrease in abundance. b Asymptomatic SOD1 (*n* = 13) compared to controls. Differentially abundant proteins were also mapped by module. Log_2_ fold change (x-axis) and one-way ANOVA with Benjamini–Hochberg corrected by disease -log10 *p*-values (y-axis). Proteins significantly (*p* < 0.05) increased in abundance are depicted in red, significantly decreased in blue, and neither in grey. c. Differentially abundant proteins from asymptomatic C9orf72 HRE versus control were compared to asymptomatic SOD1 mutation carriers in a Venn diagram. Most robustly increased and decreased proteins are shown in red and blue boxes; respectively.
Supplemental Fig. 4. a. Volcano plot representing differential protein abundance comparing SOD1 ALS (*n* = 22) versus control (*n* = 72). Log_2_ fold change (x-axis) and one-way ANOVA with Benjamini–Hochberg corrected by disease -log10 *p*-values (y-axis) are shown for each protein (*n* = 2,330). Proteins that were significantly (p ≤ 0.05) down in disease (SOD1 ALS, relative to control) are depicted in blue (*n* = 270), proteins that were significantly up are depicted in red (*n* = 186), and proteins that were neither significantly up nor down are grey. b. SOD1 protein abundance compared among all individuals without an SOD1 mutation, those with either SOD1^A5 V^ or SOD1^A5 T^ (including both symptomatic and asymptomatic carriers), and all other SOD1 mutation carriers including asymptomatic and symptomatic carriers. One-way ANOVA was used to determine if a difference was present between the SOD1-mutation groups. Note SOD1 protein is significantly reduced in those with a point mutation at position 5. c. SOD1 specific-peptide level quantification across controls and disease subgroups. Peptides were visualized for overlap of the canonical SOD1 protein sequence (P00441). Individual mutations are depicted with unique colors. Peptides in red come from DIA-MS, peptides in blue come from TMT-MS. d. Boxplots for abundance of each peptide identified were evaluated with one-way ANOVA. Each datapoint in the SOD1 ALS group is annotated with the mutation associated with each patient

## Data Availability

All raw and processed mass specrotmetry data have been deposited at Synapse.org under accession number syn53424735 and are available at the following URL: https://www.synapse.org/EmoryALS.
