# Supplementary figures and images for: Network analysis of the cerebrospinal fluid proteome reveals shared and unique differences between sporadic and familial forms of amyotrophic lateral sclerosis
Source: Mol Neurodegener. 2025 May 15;20:58. doi: 10.1186/s13024-025-00838-9 (PMC12082929; doi:10.1186/s13024-025-00838-9)

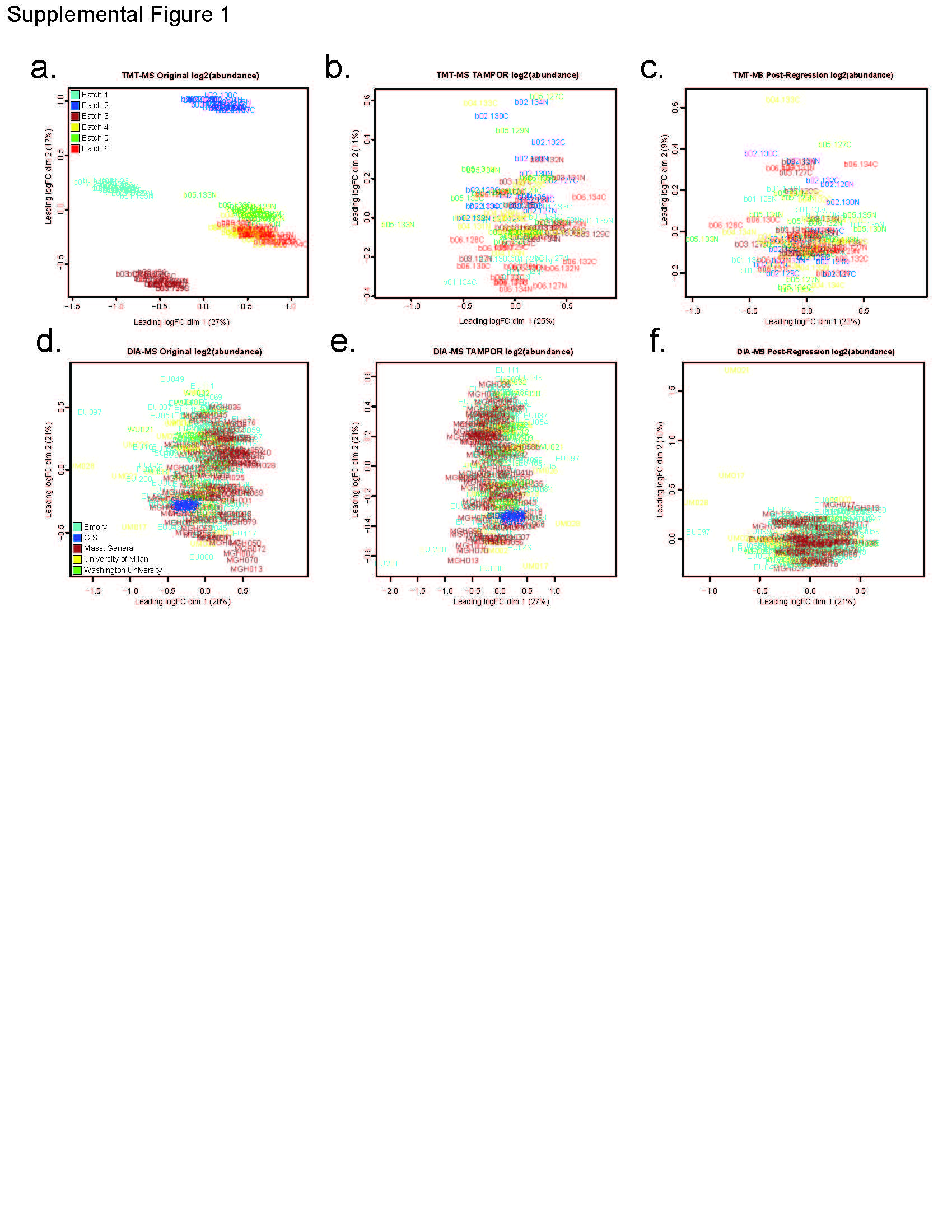

Supplement: Supplementary file 2 — Supplementary Material 2. Supplemental Fig. 1. a. Multidimensional scaling was used on raw data that had been log-transformed to visualize multidimensional proteomic distribution in two-dimensional space. b. Post-TAMPOR mode 3 distribution of TMT-MS data removes batch-level effect present in raw data. c. Post-regression TMT-MS data indicate that distribution is not affected by methodological artifacts. d. Raw DIA-MS data processed with log-transformation demonstrate the difference in unprocessed DIA and TMT-MS. e. Post-TAMPOR mode 4 distribution of individual points show a reduction in any clustering that may be due to DIA-MS. f. Post-regression distribution is further removed of methodical influence. Panels a-c demonstrate distribution of TMT-MS proteome and panels and d-f demonstrate distribution of DIA-MS. Each datapoint represents an individual tissue sample with colors indicating, in TMT-MS, shared batch and, in DIA-MS, shared center of origin. Supplemental Fig. 2. a. Shared and diverging differentially abundant proteins from single center TMT-MS quantification were compared between sporadic ALS and C9orf72 ALS. This scatterplot includes cases from the Emory single center dataset. Only proteins that were differentially abundant in both ALS subtypes are visualized. The number of proteins in each quadrant is denoted by “n”. b. Volcano plot showing differential abundance profiles comparing asymptomatic C9orf72 ALS (n = 10) and sporadic ALS (n = 35). Proteins that were significantly (p ≤ 0.05) down in disease (C9orf72 ALS, relative to control) are depicted in blue (n = 151), proteins that were significantly up are depicted in red (n = 139), and proteins that were neither significantly up nor down are grey. Supplemental Fig. 3. a. Volcano plot showing differential abundance profiles comparing asymptomatic C9orf72 carriers (n = 59) and controls (n = 72). Differentially abundant proteins were mapped by module. The height of the bars represents the fraction of module m [file 13024_2025_838_MOESM2_ESM.zip › Trautwig, et al supp fig 1.jpg]

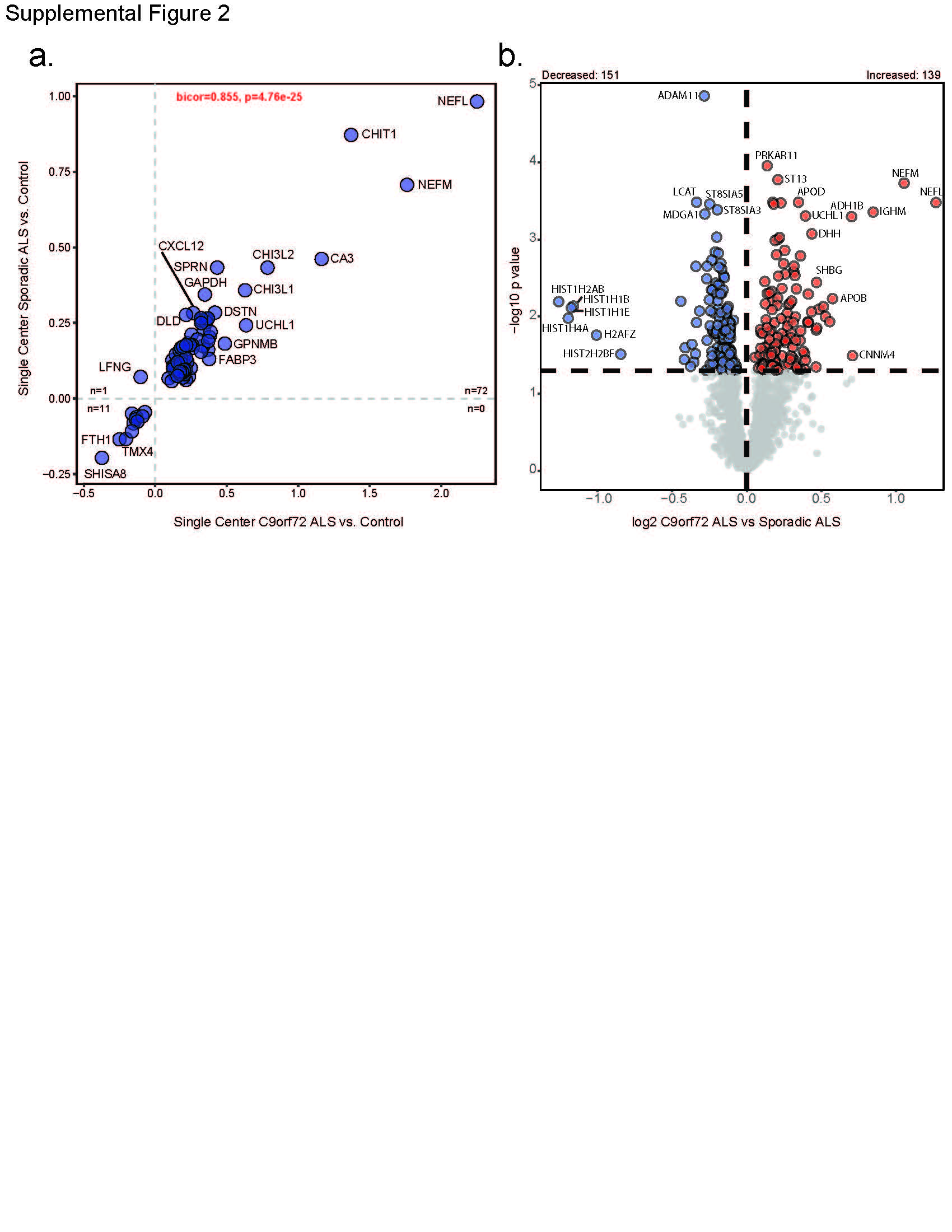

Supplement: Supplementary file 2 — Supplementary Material 2. Supplemental Fig. 1. a. Multidimensional scaling was used on raw data that had been log-transformed to visualize multidimensional proteomic distribution in two-dimensional space. b. Post-TAMPOR mode 3 distribution of TMT-MS data removes batch-level effect present in raw data. c. Post-regression TMT-MS data indicate that distribution is not affected by methodological artifacts. d. Raw DIA-MS data processed with log-transformation demonstrate the difference in unprocessed DIA and TMT-MS. e. Post-TAMPOR mode 4 distribution of individual points show a reduction in any clustering that may be due to DIA-MS. f. Post-regression distribution is further removed of methodical influence. Panels a-c demonstrate distribution of TMT-MS proteome and panels and d-f demonstrate distribution of DIA-MS. Each datapoint represents an individual tissue sample with colors indicating, in TMT-MS, shared batch and, in DIA-MS, shared center of origin. Supplemental Fig. 2. a. Shared and diverging differentially abundant proteins from single center TMT-MS quantification were compared between sporadic ALS and C9orf72 ALS. This scatterplot includes cases from the Emory single center dataset. Only proteins that were differentially abundant in both ALS subtypes are visualized. The number of proteins in each quadrant is denoted by “n”. b. Volcano plot showing differential abundance profiles comparing asymptomatic C9orf72 ALS (n = 10) and sporadic ALS (n = 35). Proteins that were significantly (p ≤ 0.05) down in disease (C9orf72 ALS, relative to control) are depicted in blue (n = 151), proteins that were significantly up are depicted in red (n = 139), and proteins that were neither significantly up nor down are grey. Supplemental Fig. 3. a. Volcano plot showing differential abundance profiles comparing asymptomatic C9orf72 carriers (n = 59) and controls (n = 72). Differentially abundant proteins were mapped by module. The height of the bars represents the fraction of module m [file 13024_2025_838_MOESM2_ESM.zip › Trautwig, et al supp fig 2.jpg]

Supplemental Figure 3

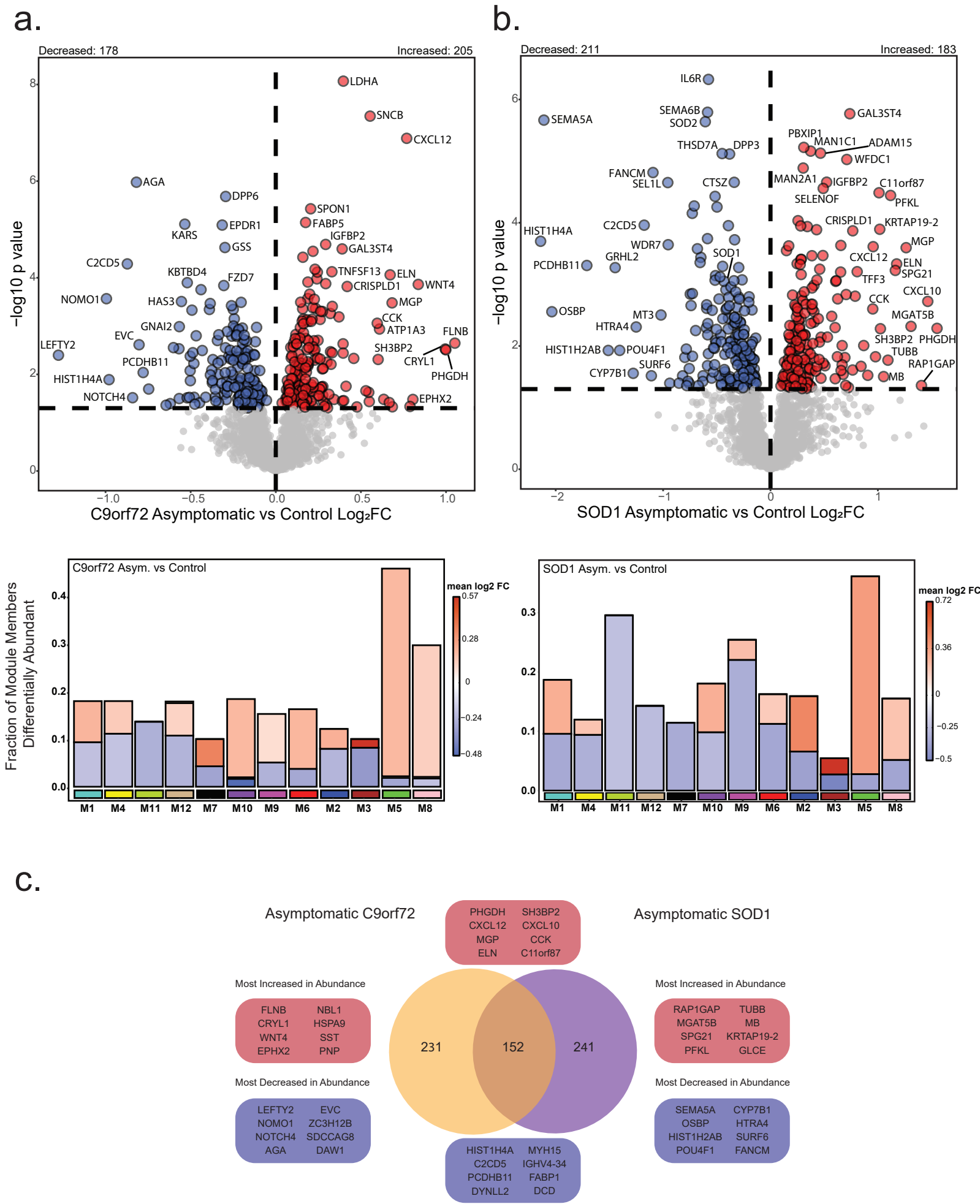

Supplement: Supplementary file 2 — Supplementary Material 2. Supplemental Fig. 1. a. Multidimensional scaling was used on raw data that had been log-transformed to visualize multidimensional proteomic distribution in two-dimensional space. b. Post-TAMPOR mode 3 distribution of TMT-MS data removes batch-level effect present in raw data. c. Post-regression TMT-MS data indicate that distribution is not affected by methodological artifacts. d. Raw DIA-MS data processed with log-transformation demonstrate the difference in unprocessed DIA and TMT-MS. e. Post-TAMPOR mode 4 distribution of individual points show a reduction in any clustering that may be due to DIA-MS. f. Post-regression distribution is further removed of methodical influence. Panels a-c demonstrate distribution of TMT-MS proteome and panels and d-f demonstrate distribution of DIA-MS. Each datapoint represents an individual tissue sample with colors indicating, in TMT-MS, shared batch and, in DIA-MS, shared center of origin. Supplemental Fig. 2. a. Shared and diverging differentially abundant proteins from single center TMT-MS quantification were compared between sporadic ALS and C9orf72 ALS. This scatterplot includes cases from the Emory single center dataset. Only proteins that were differentially abundant in both ALS subtypes are visualized. The number of proteins in each quadrant is denoted by “n”. b. Volcano plot showing differential abundance profiles comparing asymptomatic C9orf72 ALS (n = 10) and sporadic ALS (n = 35). Proteins that were significantly (p ≤ 0.05) down in disease (C9orf72 ALS, relative to control) are depicted in blue (n = 151), proteins that were significantly up are depicted in red (n = 139), and proteins that were neither significantly up nor down are grey. Supplemental Fig. 3. a. Volcano plot showing differential abundance profiles comparing asymptomatic C9orf72 carriers (n = 59) and controls (n = 72). Differentially abundant proteins were mapped by module. The height of the bars represents the fraction of module m [file 13024_2025_838_MOESM2_ESM.zip › Trautwig, et al Supp Fig 3.pdf]

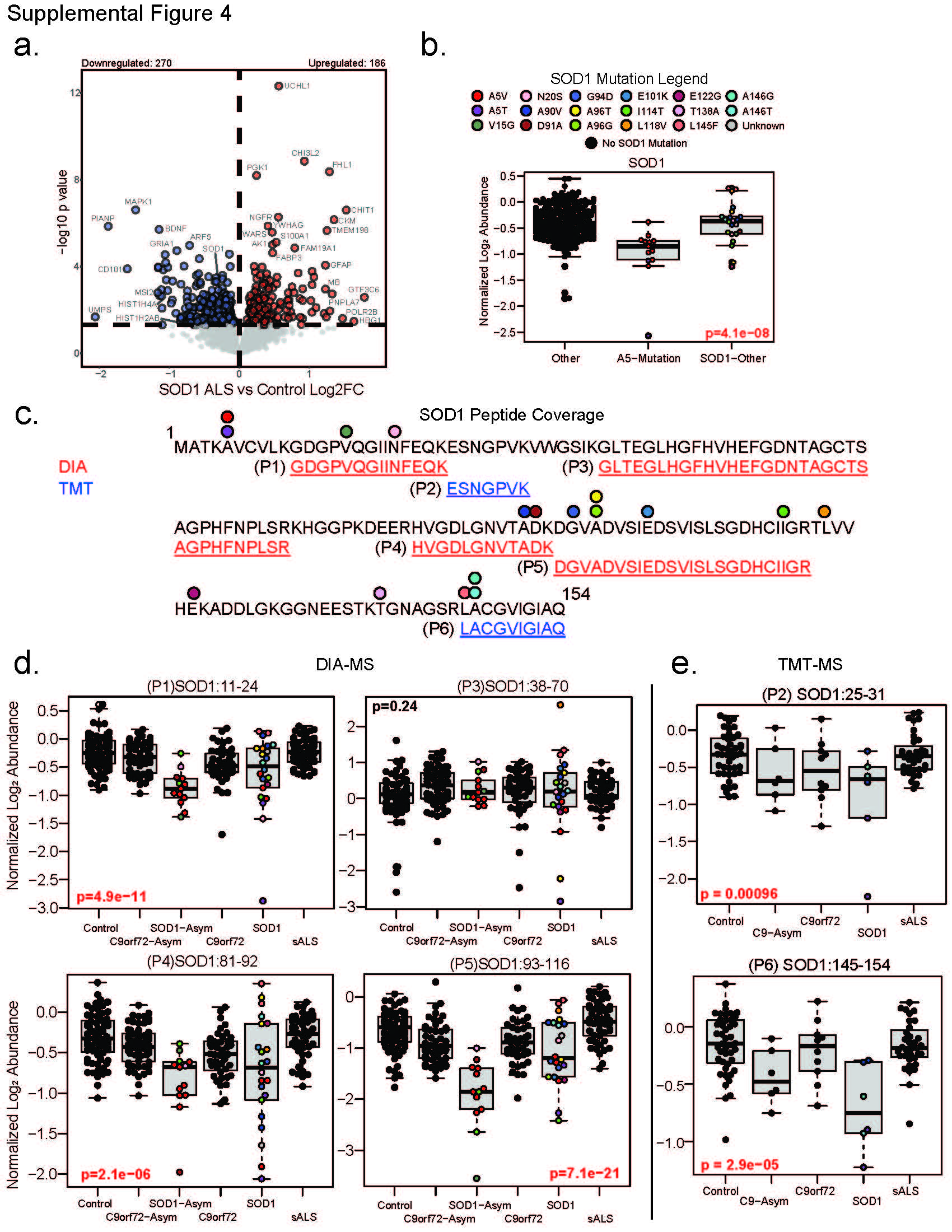

Supplement: Supplementary file 2 — Supplementary Material 2. Supplemental Fig. 1. a. Multidimensional scaling was used on raw data that had been log-transformed to visualize multidimensional proteomic distribution in two-dimensional space. b. Post-TAMPOR mode 3 distribution of TMT-MS data removes batch-level effect present in raw data. c. Post-regression TMT-MS data indicate that distribution is not affected by methodological artifacts. d. Raw DIA-MS data processed with log-transformation demonstrate the difference in unprocessed DIA and TMT-MS. e. Post-TAMPOR mode 4 distribution of individual points show a reduction in any clustering that may be due to DIA-MS. f. Post-regression distribution is further removed of methodical influence. Panels a-c demonstrate distribution of TMT-MS proteome and panels and d-f demonstrate distribution of DIA-MS. Each datapoint represents an individual tissue sample with colors indicating, in TMT-MS, shared batch and, in DIA-MS, shared center of origin. Supplemental Fig. 2. a. Shared and diverging differentially abundant proteins from single center TMT-MS quantification were compared between sporadic ALS and C9orf72 ALS. This scatterplot includes cases from the Emory single center dataset. Only proteins that were differentially abundant in both ALS subtypes are visualized. The number of proteins in each quadrant is denoted by “n”. b. Volcano plot showing differential abundance profiles comparing asymptomatic C9orf72 ALS (n = 10) and sporadic ALS (n = 35). Proteins that were significantly (p ≤ 0.05) down in disease (C9orf72 ALS, relative to control) are depicted in blue (n = 151), proteins that were significantly up are depicted in red (n = 139), and proteins that were neither significantly up nor down are grey. Supplemental Fig. 3. a. Volcano plot showing differential abundance profiles comparing asymptomatic C9orf72 carriers (n = 59) and controls (n = 72). Differentially abundant proteins were mapped by module. The height of the bars represents the fraction of module m [file 13024_2025_838_MOESM2_ESM.zip › Trautwig, et al supp fig 4.jpg]
